# Jumping on the Bandwagon: The Role of Voters’ Social Class in Poll Effects in the Context of the 2021 German Federal Election

**DOI:** 10.1007/s11615-022-00417-3

**Published:** 2022-08-10

**Authors:** Fabienne Unkelbach, Melvin John, Vera Vogel

**Affiliations:** 1 Chair of Consumer and Economic Psychology, University of Mannheim, A5, 6, 68159 Mannheim, Germany; 2 Chair of Microsociology and Social Psychology, University of Mannheim, Mannheim, Germany; 3 Mannheim Centre for European Social Research (MZES), University of Mannheim, Mannheim, Germany

**Keywords:** Mass media, Social influence, Social psychology, Voting intention, Rolling cross-section study, Massenmedien, Sozialer Einfluss, Sozialpsychologie, Wahlabsicht, Rolling Cross-Section Wahlkampfstudie

## Abstract

Published findings of opinion polls are an important part of the political coverage before elections. Thus, researchers have long investigated whether the perceived popularity of political parties can lead to even more voters following this majority. However, empirical findings on this so-called political bandwagon effect are mixed. In the present paper, we integrate theories from political science and social psychology to explain these inconsistencies through social class as a potential moderating variable. Based on previous findings regarding consumer decisions, we hypothesized that bandwagon effects are greater among voters with lower social class. To investigate this hypothesis, we combined data from the German Longitudinal Election Study (GLES) Rolling Cross-Section 2021, which was collected over the 55 days before the 2021 German federal election, with the results of published preelection polls. Using separate multilevel models for each of the parties, we found no evidence for bandwagon effects. Only for the Social Democratic Party were poll results related to voting intentions assessed on the following day, suggesting that polls might have contributed to the party’s electoral success. However, there was no evidence for a moderation of bandwagon effects by voters’ social class. Accordingly, we could not resolve the mixed findings in this field of research. Our results point to important open questions in research on bandwagon effects in multiparty systems as well as on effects of social class in Germany.

**Supplementary Information:**

The online version of this article (10.1007/s11615-022-00417-3) contains supplementary material, which is available to authorized users.

## Abstract (de)

Ergebnisse von Meinungsumfragen sind ein wichtiger Bestandteil der politischen Berichterstattung vor Wahlen. Daher wird seit Langem untersucht, ob die aufgrund von Umfrageergebnissen wahrgenommene Popularität politischer Parteien dazu führen kann, dass sich Wählende der scheinbaren Mehrheit anschließen. Die empirischen Ergebnisse zu diesem sogenannten politischen Bandwagon-Effekt sind jedoch gemischt. Im vorliegenden Artikel haben wir Theorien aus der Politikwissenschaft und der Sozialpsychologie integriert, um diese Unstimmigkeiten durch den sozialen Status als mögliche moderierende Variable zu erklären. Auf der Grundlage früherer Erkenntnisse zu Konsumentscheidungen stellten wir die Hypothese auf, dass Bandwagon-Effekte bei Wählenden mit niedrigerem sozialem Status größer ausfallen. Zur Untersuchung dieser Hypothese kombinierten wir Daten der German Longitudinal Election Study (GLES) Rolling Cross-Section-Wahlkampfstudie 2021, die in den letzten 55 Tagen vor der Bundestagswahl 2021 durchgeführt wurde, mit Umfrageergebnissen, die in diesem Zeitraum veröffentlicht wurden. In separaten Mehrebenenmodellen für jede der Parteien zeigte sich keine Evidenz für Bandwagon-Effekte. Lediglich für die SPD standen die Umfrageergebnisse in Zusammenhang mit der am Folgetag berichteten Wahlabsicht, was darauf hindeutet, dass die Umfragen zum Wahlerfolg der Partei beigetragen haben könnten. Wir fanden jedoch keine Evidenz für eine Moderation der Bandwagon-Effekte durch den sozialen Status der Wählenden. Dementsprechend konnten wir die gemischten Ergebnisse in diesem Forschungsfeld nicht auflösen. Unsere Ergebnisse weisen auf wichtige offene Fragen in der Forschung zu Bandwagon-Effekten in Mehrparteiensystemen sowie zu Effekten des sozialen Status in Deutschland hin.

## Introduction

In modern democracies and especially in times of elections, people constantly have the opportunity to get information from the mass media on others’ political opinions and voting preferences. One of the most important sources of such information consists of published findings from public opinion polls (Moy and Rinke 2012). Over the past decades, polls have become an increasingly important part of the political coverage (Brettschneider 2008). For example, in the last 5 weeks before the German federal election in 2013, the proportion of people who reported to have paid attention to such polls rose drastically and reached approximately 70% just before the election (Partheymüller and Schäfer 2013). This trend is related to a pervasive tendency toward so-called horse-race journalism, which focuses more on the question of who’s ahead than on substantive issues during the election campaign (Genz et al. 2001). While the great presence of polls in the media has long been critically debated, it became especially controversial during the 2021 German federal election campaign when a polling institute reported projections in the preelection phase including data from postal voters on their already cast votes (Thiel 2021).

Different kinds of poll effects have been distinguished. For example, becoming aware of public opinion via polls can elicit *strategic voting* behavior (i.e., people vote for a party/candidate because of strategic reasons even though it is not their preferred choice) as well as the so-called *underdog effect* (i.e., people favor parties/candidates who are behind in polls) (Moy and Rinke 2012). Furthermore, it has been argued that the perceived popularity of political parties and candidates can result in the so-called *political bandwagon effect* (Moy and Rinke 2012). This refers to the phenomenon in which some people tend to follow the perceived majority and vote for candidates, parties, or political opinions that are ahead in the polls (Schmitt-Beck 2015). Thus, bandwagon effects can be understood as an instance of majority influence in the political context.

In the present research, we argue that voters’ social class is a possible moderator variable of bandwagon effects. Recent findings from social psychology suggest that majority influence is stronger among individuals with lower (vs. higher) social class in other choice contexts (Na et al. 2016; Stephens et al. 2011). By transferring this finding to the political context, we investigated whether voters’ social class moderates the effect of a majority party preference (as perceived via public poll results) on voters’ own vote intention, i.e. the political bandwagon effect.

We examine this research question in the context of the 2021 German federal election because it provides an extraordinarily promising context to investigate poll effects for several reasons. First, this was the first federal election in the postwar history of Germany without the incumbent chancellor running. Due to this political change, preelection poll results were characterized by great dynamics before the 2021 election. This is a prerequisite for adequate statistical power to examine the existence of a bandwagon effect and voters’ social class as its assumed boundary condition. For example, in August 2021 the Social Democratic Party (SPD) was ahead of the Christian Democratic Union (CDU) in poll results for the first time in almost 15 years (Grahn and Süßmann 2021). Second, the COVID-19 crisis has made existing social disparities especially salient. Contradicting its initially assumed role as a “great equalizer” (Cuomo 2020), studies suggest that the pandemic has instead increased existing social inequalities by affecting people of different social classes to a different extent (Bundeszentrale für politische Bildung 2021). Based on data from the German Longitudinal Election Study (GLES) Rolling Cross-Section 2021 (GLES 2022), the present research makes an important contribution to a) the controversy about polls as an integral part of the political news coverage and b) the profound understanding of consequences of social class differences.

## Theoretical Argument, Literature Review, and Hypothesis

### Majority Influence in the Political Context: The Political Bandwagon Effect

In the political context, voters do not only develop their own voting intention but also form an impression of others’ preferences. Importantly, beliefs about the general electorate’s party support are influenced by polls spread by the mass media and have the potential to influence individuals’ voting preferences (e.g., Moy and Rinke 2012). Poll effects based on the tendency to follow a perceived majority opinion have often been investigated under the term bandwagon effect. It especially refers to the influence of preelection polls on individuals’ voting preferences in the sense that support for the view presented as being favored by a majority of society increases (Barnfield 2020; Schmitt-Beck 2015). Thus, bandwagon effects can affect individuals’ attitudes toward political issues, parties, and candidates as well as actual voting behavior (Moy and Rinke 2012). Thereby, polls constitute the most visible signal of majority support. A consequence of bandwagon effects is that the perceived public opinion can turn into a self-fulfilling prophecy (Schmitt-Beck 2015).

Different theoretical accounts have been used to explain why voters jump on the bandwagon (for an overview, see Hardmeier 2008). In the following, we will briefly highlight the most influential approaches. From a social psychological perspective, bandwagon effects have often been understood as a manifestation of *conformity. *In his seminal study, Asch (1956) found that individuals conform to the opinion of a majority of people surrounding them even when the majority position is clearly incorrect. Advancing these findings, studies showed that the perception that a majority of others evaluate something positively leads to individuals evaluating this attitude or object more positively, too (e.g., Erb et al. 1998). People thus follow a consensus heuristic that implies that what a majority is doing must be the preferable option (Erb and Bohner 2010).

Aside from psychological concepts used to explain the mechanisms of bandwagon effects, research from political science has long emphasized the role of so-called *impersonal influence *(Mutz 1998). This construct describes the effect of information about the beliefs of collectives of others who are not part of an individual’s personal contacts. In this regard, bandwagon effects have long been tied to a so-called gratification mechanism that refers to voters switching to the “winning side” solely because of the expected gratification of belonging to the “winners” (Mutz 1998).

Even though many potential causal mechanisms and conceptualizations of majority influence have been discussed, there is still a lack of empirical evidence for a conclusive model of political bandwagon effects (Schmitt-Beck 2015). Indeed, it is conceivable that the concept of bandwagon effects is not inherently linked with one of the proposed mechanisms but that different mechanisms might be in play (Barnfield 2020).

Empirical research on bandwagon effects includes a variety of studies that differ in their design and political setting. However, there are two main aspects that can be used to categorize these studies (for an overview, see Barnfield 2020). First, bandwagon effects have been investigated with regard to two outcomes: the switch in vote choice from one alternative to another (conversion) and a decision to turn out to vote (mobilization) (Barnfield 2020; Morton et al. 2015). Because most previous studies have investigated bandwagon effects on conversion, we will also focus on these effects when speaking of bandwagon effects.

Second, studies on bandwagon effects have differed in their independent variable—the aspect of opinion polls that influences voters’ preferences. While there is some evidence for the effect of a rise of public support of a candidate’s respective party from one time point to the second one (dynamic bandwagon effects; e.g., van der Meer et al. 2016), most research has focused on the (leading) position in poll results of one party in comparison to others at one point in time (static bandwagon effects; e.g., Schmitt-Beck 1996).

In addition to these conceptual differences, the political context plays an important role in investigations on bandwagon effects. The concept of leading in the polls is well applicable to first-past-the-post systems like that of the United States, and most evidence on bandwagon effects stems from presidential primaries in the United States (e.g., Callander 2007). But what does success/leading in the polls mean in the German multiparty context with proportional representation? Recent research suggests that the definition of success, or of being a “winner,” in opinion polls is more ambiguous in multiparty contexts with proportional representation systems (cf. Barnfield 2020). Due to the common formation of coalition governments, “winning” an election is not limited to being the party with the largest vote share (Meffert et al. 2011). Recent research in proportional representation contexts has emphasized that there are several aspects that can lead to a party’s being portrayed and perceived as a winner, e.g., exceeding expectations, getting the largest number of seats, overcoming the electoral threshold (Stolwijk et al. 2016), or being predicted with high certainty to be part of a government (Riambau 2018). Thus, both large and small parties might justifiably claim to be successful in their election campaigns (Hardmeier and Roth 2003). Accordingly, we investigated potential poll effects for all major German parties to be able to get a complete picture.

In the German context, there is first-hand evidence that interest in political media coverage a) influences voters’ expectations regarding the election outcome and b) consequentially leads to voters choosing the party that was leading in the polls in the 1990 national election (Schmitt-Beck 1996). Similar results could be found for the 2005 parliamentary election in a study based on data from a rolling cross-section (RCS) study (Faas et al. 2008). However, a reanalysis of the GLES RCS data for the 2005 election with a different analysis approach^1^ suggested that the polls had an influence only on voting turnout intention and coalition expectations but not on voting intentions for the different parties (Hoffmann and Klein 2013). Accordingly, we concluded that further investigations on poll effects in the German context were necessary.

Whereas previous research has explained the mixed empirical evidence for the political bandwagon effect through methodological issues and a lack of conceptual clarity (cf. Barnfield 2020), we argue that a third reason might play a role: Potential moderating variables of majority influence could influence the size of the political bandwagon effect. Importantly, recent social psychological findings suggest that a sociodemographic characteristic of individuals, namely their social class, is related to the tendency to follow a perceived majority. We, thus, focused on this variable as a potential moderator of bandwagon effects in the present research.

### Social Class and Susceptibility to Social Influence

In the last decade, psychological perspectives on social class have been developed: a so-called social cognitive perspective (Kraus et al. 2012) and a cultural approach (Stephens et al. 2014). Whereas the first one focuses on the way different material resources can influence basic psychological tendencies, the second one is based on the notion that people’s social class is an important determinant for the sociocultural contexts in which people spend most of their lives. Within these frameworks, objective components of social class (objective socioeconomic status [SES]) refer to a person’s level of access to (material) resources and are often measured via one’s educational attainment and financial means, as well as occupational prestige (Oakes and Rossi 2003). By contrast, subjective components of social status are defined as a person’s perception of their relative standing in society, which is derived from the comparison of one’s material wealth to those of others (e.g., Kraus et al. 2012, 2017). It is assumed that objective SES and subjective social status (SSS) constitute important factors of social class contexts that influence people’s experiences of being from a certain social class background and refer to different aspects of social class (Adler et al. 2000; Kraus et al. 2012). Importantly, research from both theoretical approaches on social class shows that the material conditions in which people are raised and live influence a range of psychological and behavioral outcomes (Kraus et al. 2012; Manstead 2018).

First, people of lower and higher social class differ in their self-concept (Kraus et al. 2012; Manstead 2018). A self-concept is defined as a person’s thoughts, beliefs, and feelings about the self as an object (Markus and Kitayama 1991, 2010). More concretely, there is first evidence that people of lower social class are more likely to develop an *interdependent* self (Grossmann and Varnum 2011). This means that they tend to focus on their self as embedded in social relationships, a focus that is often explained by their experience of constrained opportunities for making free life choices. In contrast, people of a higher social class are more likely to develop an *independent* self-concept (Kraus et al. 2012). This means that they tend to see themselves as separate, unique entities, and this tendency has often been explained by their larger freedom to make life decisions according to their own interests and desires.

Second, these social class differences in self-concepts are reflected in the way people make choices under social influence. Individuals from lower social classes are relatively more likely to make choices that promote similarity to and connection with others, whereas individuals from higher social classes are relatively more likely to make choices that produce uniqueness and differentiation from others (Na et al. 2016; Stephens et al. 2007, 2011). For example, it was found that participants with lower objective SES (as classified via their parents’ educational attainment) more often chose the same product as an ostensible former participant and liked their chosen product more when another person apparently made the same choice (vs. a different choice) (Stephens et al. 2007, studies 2 and 3). However, the choice of another person had no influence on the ratings of the chosen product for participants with higher objective SES.

Additionally, people of lower social class are more susceptible to a perceived majority preference when it comes to product choices (Na et al. 2016). It could be demonstrated that mainly participants of lower social class aligned their product choices with a majority’s preference even when that choice contradicted their personal preferences (Na et al. 2016, study 1). Importantly, this effect was found using objective SES as well as SSS. First evidence suggests that these social class differences in the sensitivity to preferences of others are indeed mediated by their independent vs. interdependent self-concepts (Na et al. 2016, studies 2 and 3).

Thus, whereas majority influence has often been considered a well-established phenomenon, recent studies suggest that these effects cannot be found uniformly across all social classes. In the present research, we transfer these findings to the political context and, more concretely, to the political bandwagon effect.

### Current Research: The Role of Social Class in Bandwagon Effects

Even though multiple studies on bandwagon effects have been conducted over the past decades, empirical evidence is mixed. One potential explanation for these inconsistencies might be the presence of moderator effects. While the political system has often been considered an important context variable, few studies so far have focused on voters’ characteristics as moderators of the size of bandwagon effects. This, however, is important not only to better understand the relevance of bandwagon effects for voters’ behavior but also to arrive at a better understanding of poll influences overall.

In the present research, we argue that recent social psychological findings on the relationship between people’s social class and their tendency to follow a majority can offer new insights into boundary conditions of bandwagon effects. More concretely, people with lower objective SES and SSS have been found to be more likely to align their product choices with social preferences (Na et al. 2016; Stephens et al. 2011). Research suggests that this can be explained by an interdependent self-concept and a stronger focus on external factors when making decisions (Kraus et al. 2012; Na et al. 2016). However, the relationship between social class and the tendency to follow a majority has so far been investigated only in product choice settings.

Based on these findings, we argue that voters’ social class also plays a role in the political context, specifically in the formation of voting intentions. First evidence that this transfer is possible stems from research on environmental concerns. It has been demonstrated that perceived descriptive social norms about pro-environmental behavior, i.e., the perception of how a majority of others behave, are more predictive of support for pro-environmental action among individuals with lower objective (vs. higher) SES (Eom et al. 2018).

Furthermore, there is empirical evidence that the political bandwagon effect is stronger among less educated voters (Schmitt-Beck 1996), whereas educational attainment can be seen as a proxy for objective SES. This result suggests that lower-class voters correctly understand the information conveyed in polls but use it systematically differently than higher-class voters do. At the same time, this finding contradicts the potential alternative assumption that people of lower social class attach less weight to poll results when making their own voting decisions because of their lower level of generalized trust (Dahlhaus and Schlösser 2021; Kim et al. 2022). Based on these assumptions and results, we derived the hypothesis that the results of preelection polls more strongly influence voting intentions among people of lower (vs. higher) social class. We investigated this hypothesis in the context of the 2021 German parliamentary election. Our research can thereby produce further insights into bandwagon effects in multiparty systems and proportional representation systems. Because of the interdisciplinary approach and the use of suitable data from the 2021 GLES RCS (Faas et al. 2008; Hoffmann and Klein 2013), our research expands the literature on bandwagon effects and on social cognitive effects of social class in several ways: By clarifying a boundary condition of the political bandwagon effect, the present research contributes to understanding the size and relevance of effects of preelection polls on political attitudes and voting behavior. Thus, it has the potential to inform the debate on regulations on the publication of these polls. At the same time, our study goes beyond previous social psychological research on social class by investigating these effects in the election context. More concretely, our research offers new insights into the generalizability of social cognitive effects of social class, which have so far only been found in limited contexts. Additionally, it allows us to investigate boundary conditions of conformity, a central social psychological concept.

Combining both strands of research makes it possible to investigate the generalizability of the relationship between social class and the tendency to follow a majority. In the long run, our findings might help prepare the ground for developing methods to enhance the political information processing of people from different social-class backgrounds.

## Data and Methods

We preregistered all our analyses on the open science framework. The preregistration can be accessed on osf.io (https://osf.io/g6r7v/).

### Data

We examined the moderating effect of social class on the relationship between the perceived majority intention and individual voting intention based on data from the GLES RCS 2021.^2^ The RCS is a large-scale, cross-sectional survey based on phone interviews. It comprises German-speaking respondents living in private households who have a landline telephone connection or a mobile number within the Federal Republic of Germany and were eligible to vote in the federal election of 2021 (GLES 2022). Adjustment weights were included based on sociodemographic characteristics (i.e., gender, age, and education) as well as regional characteristics (e.g., municipal regions, east–west comparison; for detailed information on the survey design, see GLES 2022).

Specifically, we used data from the preelection survey collected before the German parliamentary election between August 2, 2021, and September 25, 2021. For each of the 55 field days, the goal was to realize 130 interviews. This resulted in a total sample size of 7068 respondents. To ensure that there were no missing data at the construct level, we listwise excluded all cases with missing data on the focal measures (i.e., educational attainment and current gainful employment status as indicators of objective social class, individual voting intention [and the actual majority’s voting intention]).

The RCS data are highly suitable for our research question for three reasons. First, the RCS covers a relatively long time span before the German election and captures voting intentions. Second, the RCS comprises all relevant information for our research question: respondents’ educational attainment and their current gainful employment status as proxy for social class, respondents’ voting intention, and respondents’ perception of poll results in the previous week, i.e. the perception of a majority’s voting intention. Third, the RCS includes data from a random sample of the population described above on each field day (GLES 2022).

To examine the potential influences of poll results (i.e., the current majority’s voting intention) on individual voting intentions, we combined the RCS data with results of published preelection polls (Blais et al. 2006; Faas et al. 2008). This procedure made it possible to investigate influences of published poll results on voting intentions on a daily basis over the last 8 weeks of the election campaign before the 2021 German federal election (Faas et al. 2008). We used data from the eight leading polling institutes in Germany (in alphabetical order): Allensbach, Forsa, Forschungsgruppe Wahlen, GMS, Infratest dimap, INSA, Kantar Emnid, and Yougov. The data were obtained from Wahlrecht.de (2021).^3^ We matched each field day’s RCS data with the results of preelection polls published 1 day before the RCS field day for each of the six political parties currently represented in the German Bundestag. Because some polls were published in the evening, which could result in some respondents not having the chance to see these polls, a lag of 1 day was chosen. In the event that no new polls were published on a particular day, we matched the RCS data with the most recently published poll results. If results from two or more polling firms were published on the same day, we used the average of these results.

**Fig. 1:**
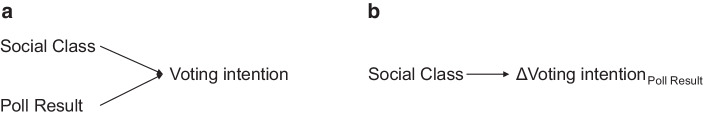
**a** Directed acyclic graph (DAG) for the main effects of social class and poll result on voting intention. **b** Interaction DAG (IDAG, Nilsson et al. 2021) for the hypothesized moderation effect. This states that social class influences the effect of poll results on voting intentions as measured in the 2021 rolling cross-section study

In Fig. 1, the assumed main effect of poll results on voting intentions is displayed as a directed acyclic graph (see Panel a). Even though we had no specific hypotheses about the main effect of social class on voting intentions, we have displayed this path in the model for the sake of completeness. One could argue that our study design only allows examination of correlations. Potentially, unobserved confounders such as external events during the investigated time span could have influenced poll results and the voting intentions measured in the RCS data. Nevertheless, this appears to be unlikely for several reasons. First, the data collection of the polling institutes took place a few days before the assessment of the RCS data. Second, each polling institute collected the poll data over a different number of days and published the data one or several days after data collection. Thus, an external event that might have influenced the poll results would have needed to exert an influence over several days. Even if this were the case, other events that happened temporally closer to the RCS data collection would have been more salient for the RCS respondents and thus would have overshadowed the effect of the first event on voting intention. Accordingly, we concluded that it would have been rather unlikely for an external event to have caused both short-term fluctuations in the respective poll result and short-term fluctuations in the matched RCS data.

Furthermore, the main interest of this paper is to investigate whether the effect of poll results on voting intentions was moderated by social class (Fig. 1b). Inferences regarding this moderation are not necessarily affected by an external event that confounds the relationship between poll results and voting intentions. Instead, a confounder would have had to influence both social class and the effect of poll results on voting intentions at the same time. We argue that such a confounder is unlikely because voters’ social class is rather stable over the investigated time span and varies mainly between, rather than within, respondents (cf. OECD 2018).

### Variables

The following variables from the 2021 GLES RCS were used:

#### Individual Voting Intention

Individuals’ voting intention was assessed by the single item: “Which party will you vote for in the federal election?” Respondents were instructed to name one political party to which they would give the second vote (i.e., the vote that determines the proportions by which the political parties are represented in the German parliament). The individual voting intention was recoded in dummy-variables for the six political parties currently represented in the German Bundestag (CDU/Christian Social Union [CSU], SPD, Alternative for Germany [AfD], Free Democratic Party [FDP], the Left, Alliance 90/the Greens). Thereby, we treated the three separate answer options “CDU/CSU,” “CDU,” and “CSU” as a joint category. The reference category was defined as the voting intention for another political party or respondents who were still undecided, who would not cast a vote, or who would cast an invalid vote.

#### Objective Socioeconomic Status

We used two variables assessed in the RCS to compute a composite score of objective SES:

We used respondents’ highest level of general education as one indicator for objective social class, as we assumed that an individual’s socioeconomic position was (at least partly) given by their educational attainment. To ensure that each level of educational attainment was equally represented, we condensed the seven answer categories of the RCS item into three levels of educational attainment based on the sampling weights in the RCS: *low* (1, 2), *medium* (3, 7), *high* (4, 5). Responses indicating “other school-leaving certificate” (code 6) were coded as missing values.

As a second indicator of objective SES, we used respondents’ current gainful employment status. Respondents indicated whether they worked full time, part time, or short time; were in vocational training/studies; or did not work in a paid job at all. We condensed the five answer categories into three levels of employment: full-time (1), part-time/short-time (2, 3), and little to no gainful employment (4, 5).

Following the procedure used in previous studies on social class (e.g., Kraus and Keltner 2009), we coded and standardized educational attainment (0 for low education, 1 for medium education, and 2 for high education) and current gainful employment status (0 for little gainful employment, 1 for part-time/short-time gainful employment, and 2 for full-time gainful employment) and created a single index of objective social class by computing a sum score.

#### Subjective Social Status

Respondents’ SSS was assessed with a single item.^4^ They were asked to indicate where they thought they stood in relation to other people in Germany on a ladder from 1 to 11.

#### Perception of the Majority’s Voting Intention

The perception of the majority’s voting intention was assessed by a single item. Respondents were asked, “Did you read or see any results of current opinion polls on the federal election in the last week?” The two response options *yes* (1) and *no* (0) were dummy-coded, with *no* as the reference category.^5^

We included the following variables assessed in the 2021 RCS as control variables:

#### Interest in Politics

Interest in politics was assessed by the single item, “Quite generally, how interested are you in politics?” The response options ranged from 1 (*very interested*) to 5 (*not at all interested*).

#### Interest in the Current Election Campaign

General interest in the current election campaign was assessed by the single item, “And how interested are you in particular in this federal election campaign?” The response options ranged from 1 (*very interested*) to 5 (*not at all interested*).

#### Party Identification

Respondents’ party identification was assessed by the single item, “In Germany, many people lean toward a particular political party for a long time, although occasionally they vote for another party. How about you, do you lean toward a particular political party?” The response options included the six political parties currently represented in the German Bundestag (CDU/CSU, SPD, AfD, FDP, the Left, Alliance 90/the Greens). We treated the answer options “CDU/CSU,” “CDU,” and “CSU” as a joint category. For each of the six parties, we created a dummy variable displaying whether a person leaned toward this party (1) or leaned toward another party/did not lean toward any party/did not know (0).

#### Issue Orientation

Respondents were asked to think about the current political situation and to indicate what they thought the most important political problem in Germany currently was. After naming the perceived most important political problem, respondents were asked to indicate which party they thought would be best able to handle this problem. The response options included the six political parties currently represented in the German Bundestag (CDU/CSU, SPD, AfD, FDP, the Left, Alliance 90/the Greens). We treated the answer options “CDU/CSU,” “CDU,” and “CSU” as one category. For each of the six parties, we created a dummy variable indicating whether the respective party was named (1) or whether the party was not named/respondent answered that all parties were equally good (0).

#### Candidate Orientation

Respondents were asked how they perceived some of the leading politicians from the six parties currently represented in the German Bundestag (A. Laschet, A. Baerbock, O. Scholz, T. Chrupalla, A. Weidel, C. Lindner, J. Wissler, D. Bartsch, A. Merkel). The response options ranged from −5 (*I do not think much of the politician at all*) to 5 (*I think a great deal of the politician*).

#### Gender

Gender was assessed by three categories: *male, female*, and *nonbinary*. Because we expected the sample size to be too small to lead to meaningful results, we excluded respondents indicating a nonbinary gender from the respective analyses. We dummy-coded the variable (0 = *male*; 1 = *female*) with male as the reference category.

#### Age

Based on the self-reported year of birth, we computed the age of the participants in years (ranging from 18 to 90 years and older).

As explained above, the RCS data were matched with external data, namely the results of polls published over the course of the election campaign (Blais et al. 2006; Faas et al. 2008; Hoffmann and Klein 2013).

#### Current Majority’s Voting Intention

The current majority’s voting intention (CMVI) was adopted from the results of the so-called Sunday question. In Germany, voting intentions are typically assessed by asking respondents what party they would choose if an election were held “next Sunday.”^6^ The published poll results are a projection of the voting intentions of the German electorate for the six political parties currently represented in the German Bundestag. We generated a variable for each of the six parties that indicated the proportion of votes this party was projected to receive. Consequently, these variables only varied between field days (level 2).

### Data Exclusion and Missing Data

We excluded participants whose postal codes indicated that they cast their vote in the Saarland because not all of the six parties that were currently represented in the Bundestag were eligible with the second vote there. Specifically, because of a formal error, the state list of the party Alliance 90/the Greens was not admitted for election in the Saarland, which means that voters could not cast their second vote for this party. Technically, this was also the case for the CDU in Bavaria and for the CSU in all federal states but Bavaria; however, due to the close cooperation between these two parties, we treated them as one in our analyses.

We did not impute incomplete or missing data and instead used listwise deletion in our analyses. Furthermore, we did not consider statistical outliers to pose a problem in our analyses because each of the variables we used had only a few answer categories. By implication, extreme answer patterns that would significantly distort our results were unlikely.

### Sampling Weights

Since we were mainly concerned with testing our hypothesis, which had to be true in any sample of participants, we did not plan to include any sample weights.

## Analysis Plan

### Statistical Models, Robustness Testing, and Model Nonconvergence

To test our hypothesis, we used multilevel logistic regressions, as the RCS survey includes data from different respondents every day in a representative cross-sectional design. Consequently, the respondents were nested in different field days. In our planned analysis, we modeled respondents as level 1 units and field days as level 2 units.

The focus of our research was on the cross-level interaction between CMVI (level 2) and social class (level 1) on voting intention. Following the recommendation by Enders and Tofighi (2007), we first centered all level 1 predictors (i.e., all variables aside from CMVI) within field days (group-mean centering).^7^ This made a meaningful interpretation of the cross-level interaction effect possible (Enders and Tofighi 2007). The CMVI as level 2 predictor was grand-mean centered. After centering, we *z*-standardized all predictors. This yielded standardized point estimates of our regression coefficients akin to those obtained through standardization in ordinary least squares regression (Snijders and Bosker 2012).

In accordance with Barr et al. (2013), we followed recommendations regarding the model complexity. We included random slopes for all predictors on level 1 and used an unstructured covariance matrix. In case the models did not converge, we first ran them with a different optimizer, which has been used in a previous version of the lme4 R package (Bates et al. 2015) (“bobyqa”). If this did not solve the problem, we simplified the model complexity by omitting the random slopes for the covariates.

The outcome, voting intention, was operationalized through a dummy variable for each of the six parties. For each party, we conducted a separate multilevel model with maximum likelihood estimates and the respective voting intention as outcome. Since each of the dependent variables was binary, the models included a logit link function. We conducted our analyses in R, using the lme4 package and lmerTest (Kuznetsova et al. 2017).

For the model estimation process, we used a multistep approach. In the first step, we estimated an intercept-only model for each party, which yielded the intraclass correlation coefficient (ICC). For each model, the ICC indicated the proportion of variance in our outcomes that was due to variation between field days (level 2 variation) (Hox et al. 2010). Our hypothesis was based on the assumption that there was variance in voting intentions between field days (which could be traced back to changes in CMVI). If the ICC reached statistical significance, we would conclude that the individual voting intention systematically varied between field days.^8^

In the second step, we estimated a model for each of the six parties in which we included CMVI as a level 2 predictor and the index of objective SES as level 1 predictor. Furthermore, we included the cross-level interaction between the CMVI (level 2) and objective social class (level 1). We expected the voting intentions of individuals to be affected by the poll results, especially for individuals of a lower social class compared with individuals of a higher social class. Thus, we expected a significant cross-level interaction as support for our hypothesis.

In a third step, we tested whether the results were robust by including the two indicators of objective SES separately instead of using the composite score. More concretely, we conducted the same analysis as outlined above but included educational attainment instead of the composite score of objective SES as predictor. For this purpose, educational attainment was contrast coded (−1 for low education, 0 for medium education, and 1 for high education). Additionally, we conducted the analysis including only gainful employment status as predictor instead of the composite score of objective SES as predictor. This variable was contrast coded (−1 for little to no gainful employment, 0 for part-time/short-time gainful employment, and 1 for full-time gainful employment).

In a fourth step, we tested the robustness of the results regarding the addition of relevant covariates. For the selection of covariates, we followed previous studies on bandwagon effects in Germany (Faas et al. 2008; Hoffmann and Klein 2013). Thus, we included standard primarily sociodemographic controls (age, gender, interest in the campaign, general interest in politics) as well as standard predictors of voting behavior in the Michigan model^9^ (cf. Campbell et al. 1960), which included party identification, candidate orientation and issue orientation. We again estimated a model for each of the six parties, but in addition to the variables we added in the second step, we also included these covariates on level 1.

Additional robustness checks were conducted to investigate the assumed causality direction. More concretely, we computed the same models as outlined above but used voting intentions from the day before the polls were published (*t* − 1) instead of voting intentions from the day after the polls were published (*t* + 1) as outcome. Based on the standardized coefficients for the main effect of poll results on voting intentions, we computed a new binary variable. This indicated the number of times that the effect of poll results on voting intentions at *t* + 1 was larger than the effect on voting intentions at *t* − 1. We conducted a binomial test to investigate whether the effect on *t* + 1 was significantly more often larger than the effect on *t* − 1. If this was the case, we interpreted the result as support for the assumed causality direction.^10^ Additionally, we investigated the interaction effect between poll results and objective social class on RCS voting intentions at *t* − 1 and at *t* + 1 following the same procedure outlined above. We expected that this interaction effect would more often be larger when the polls were published before (vs. after) the RCS voting intention had been assessed.

As a final robustness check, we included the perception of the CMVI as additional predictor on level 1 in the main analysis, as we assumed that polls could affect voting intentions only when individuals took note of them. We also included all possible two-way interactions and the three-way interaction between objective social class, CMVI, and perception of the CMVI. We assumed that the voting intentions of individuals would be more strongly affected by the poll results they reported to have seen, especially for respondents of lower objective social class.^11^

### Effect Size, Statistical Power, and Inference Criteria

Since all of the models were nested, we used likelihood ratio tests for model comparisons. We considered *p* < 0.05 as statistically significant. As our hypotheses were directional, we used one-sided tests to assess the significance of the corresponding regression coefficients. No corrections were made for multiple tests. We expected to be able to use data from an average of 130 respondents per field day for 55 field days. Previous studies on the role of social class in the tendency to follow a perceived majority found medium to large effect sizes (e.g., Na et al. 2016). However, our study differs from these studies in several aspects. Most importantly, previous research was focused on the context of product choice, whereas the aim of the present research was to transfer these findings to the political context.^12^ Thus, we anticipated a small effect size for the hypothesized cross-level interaction effect between social class and poll results on voting intentions. A power analysis using an online tool for power analysis for multilevel logistic regression (Astivia et al. 2019) that is based on the R packages lme4, simglm (LeBeau 2021) and paramtest (Hugh 2017) resulted in an estimated power > 0.90 to detect a cross-level interaction effect, with beta = 0.1 for small level 1 and level 2 effects (each with beta = 0.1) and a slope variance of 0.09.^13^

### Exploratory Analyses

The results of the following exploratory analyses are presented in the online appendix. For exploratory purposes, we conducted the same analyses as presented above but used a time lag of zero days and a time lag of 2 days when matching the published poll results with RCS data. Additionally, we conducted our main analyses as specified above using subjective social class as continuous predictor on level 1. Furthermore, we considered to conduct our main analyses as specified above using transformation and adjustment weights provided with the dataset.

To get a better understanding of psychological correlates of social class in the German context, we conducted a further explorative analysis. This could provide insights into mechanisms of the hypothesized moderation of bandwagon effects via social class. The social cognitive model of social class (Kraus et al. 2012) proposes that social class is related to different psychological tendencies when perceiving one’s social environment. Therefore, we explored whether objective/subjective social class were correlated with the tendency to “focus on the whole and less on particular details” as measured in the RCS postelection survey.

## Results

### Main Analyses

After applying the preregistered exclusion criteria, the analysis dataset consisted of 5291 respondents nested in field days. This dataset was matched with poll results (CMVI) using a time lag of 1 day for the main analyses.

To assess the impact of the nesting of the data, we specified intercept-only models for each party and computed ICCs (see Table 1). For CDU/CSU, the Left, and AfD, the ICCs were zero, indicating that there was negligible variation in the intention to vote for these parties across field days. For the remaining parties, ICCs were close to zero (SPD: ICC = 0.005; FDP: ICC = 0.024; Alliance 90/the Greens: ICC = 0.002). Although the variation across field days was rather small, we could not preclude the existence of variation in the effect of objective SES as predictor on level 1 (see Barr et al. 2013). Thus, we estimated multilevel models with objective SES, CMVI, and their cross-level interaction as predictors of voting intention for each of the six parties.

**Table 1:** Results from all intercept-only models and intraclass correlation coefficients for voting intention

	CDU/CSU			SPD			FDP			Alliance 90/the Greens			The Left			AfD		
	β		*SE*	β		*SE*	β		*SE*	β		*SE*	β		*SE*	β		*SE*
Intercept	−1.775	***	0.039	−1.538	***	0.041	−2.714	***	0.071	−1.540	***	0.038	−3.021	***	0.065	−3.157	***	0.069
*ICC*	0.000			0.005			0.024			0.002			0.000			0.000		

*N* (level 2) = 55, *N* (level 1) = 5291 for each model

*AfD *Alternative for Germany, *CDU* Christian Democratic Union, *CSU* Christian Social Union, *Est.* estimate, *FDP* Free Democratic Party, *ICC* intraclass correlation coefficient, *SE* standard error, *SPD* Social Democratic Party

*p < 0.05, **p < 0.01, ***p < 0.001

For the SPD, CMVI was positively associated with voting intention^14^: β = 0.102, *SE* = 0.041, *OR* = 1.107, 95% CI [1.022, 1.201], *p* = 0.006^15^. The hypothesized interaction effect was, however, not significant: β = 0.012, *SE* = 0.037, *OR* = 1.038, 95% CI [0.940, 1.089], *p* = 0.376. For the other parties, neither the CMVI nor the interaction effect with individuals’ objective SES reached significance.^16^ The complete results are displayed in Table 2.

**Table 2:** Results from all multilevel logistic regression models predicting voting intention for the political parties represented in the German Bundestag

	CDU/CSU			SPD			FDP			Alliance 90/the Greens			The Left			AfD		
	β		*SE*	β		*SE*	β		*SE*	β		*SE*	β		*SE*	β		*SE*
Intercept	−1.789	***	0.040	−1.581	***	0.041	−2.749	***	0.074	−1.583	***	0.04	−3.027	***	0.066	−3.161	***	0.074
CMVI	0.037		0.039	0.102	**	0.041	0.021		0.071	0.038		0.039	0.094		0.065	−0.067		0.069
SES	−0.174	***	0.040	−0.343	***	0.037	0.276	***	0.066	0.356	***	0.039	−0.052		0.065	−0.010		0.076
CMVI × SES	−0.038		0.040	0.012		0.037	0.058		0.063	−0.059		0.039	−0.041		0.064	0.048		0.070

*N* (level 2) = 55, *N* (level 1) = 5291 for each model. The reported *p*-values for CMVI and the interaction are one-sided due to the directed hypotheses

*AfD *Alternative for Germany, *CDU* Christian Democratic Union, *CSU* Christian Social Union, *CMVI* current majority’s voting intention, *FDP* Free Democratic Party, *SE* standard error, *SES* objective socioeconomic status, *SPD* Social Democratic Party

*p < 0.05, **p < 0.01, ***p < 0.001

### Robustness Checks

As indicated by preregistration, we conducted further analyses to assess the robustness of our results. First, we varied our operationalization of objective SES by evaluating gainful employment and educational attainment separately instead of using a combined index. We found that our results did not change (see online attachment, Tables A1 and A2).^17^ The CMVI was not related to voting intention (except in the case of the SPD), nor did gainful employment moderate this relationship. However, we found that education moderated the relationship between CMVI and measured voting intention (lag 1) for Alliance 90/the Greens such that the relationship was stronger among less educated people. Interestingly, the relationships of employment and education with measured voting intention differed in intensity and went in opposite directions in the case of the Left and the AfD. This could indicate that the index for objective SES based on employment and education had a reduced reliability.

Second, we included covariates (gender, age, general interest in politics, campaign interest, party identification, issue orientation, and candidate orientation) in the main analyses. With the exception of objective SES, we again found no significant changes to our main results (see Table A3).

To investigate the direction of the proposed moderated effect, we matched the RCS data with polls published a day after the respective field day (see Table A4). For half of the parties (SPD, FDP, the Left), the relationship of CMVI and voting intentions from the day after the polls were published (main analyses; expected temporal direction) was stronger than the relationship of CMVI and voting intentions from the day before the polls were published. The binomial test did not reach significance: *P* (effect in expected temporal direction > effect in contrary temporal direction) = 0.500, *p* = 0.500. For the interaction effects, the pattern was similar; the standardized coefficients were larger for the expected temporal direction for three of the parties (SPD, FDP, AfD). Again, this was not significant: *P* (effect in expected temporal direction > effect in contrary temporal direction) = 0.500, *p* = 0.500. Thus, these results did not sufficiently support the assumed direction of effects. We will further discuss the implications of this finding below.

When including the reported perception of CMVI as well as all possible two-way interactions and the three-way interaction between objective SES, CMVI, and CMVI perception, the pattern of results did not change for the relationship of CMVI and voting intention or its interaction with objective SES (see Table A5). Additionally, the three-way interaction did not reach significance for any of the parties. This indicates that voting intentions were not related more strongly with the perceived CMVI for respondents with lower objective SES. However, supporting the notion of a poll effect for the SPD, we found a significant interaction between CMVI and CMVI perception: β = 0.091, *SE* = 0.041, *OR* = 1.095, 95% CI [1.011, 1.184], *p* = 0.014. This result suggests that the positive relationship between CMVI and voting intention for the SPD was greater when polls were perceived earlier than the respective field day.

### Exploratory Results

Instead of a time lag of 1 day, other time lags can be used to investigate bandwagon effects. Thus, we first computed the main analyses again for a time lag of zero days, which implies investigating the effects of poll results published on the same day as the collection of the matched RCS data. Paralleling the results for a time lag of 1 day, the relationship of poll results and voting intention remained significant for the SPD: β = 0.109, *SE* = 0.041, *OR* = 1.115, 95% CI [1.029, 1.209], *p* = 0.004. We did not find a significant interaction between objective SES and CMVI for any of the parties.

Additionally, we computed the same analyses for a time lag of 2 days, which means that we investigated the effects of CMVI published 2 days before the assessed voting intention. Again, CMVI was significantly associated with voting intention for the SPD—β = 0.105, *SE* = 0.040, *OR* = 1.111, 95% CI [1.025, 1.203], *p* = 0.005—whereas the interaction between objective SES and CMVI did not reach significance for any of the parties. The complete results for the analyses using a time lag of zero and two are presented in the online supplement (Table A6).^18^

Investigating SSS instead of objective SES led to the same pattern of results (see Table A7). The CMVI and voting intention remained significantly associated for the SPD—β = 0.082, *SE* = 0.046, *OR* = 1.085, 95% CI [0.992, 1.189], *p* = 0.038—whereas the interaction between SSS and CMVI did not reach significance for any of the parties.

We further explored whether objective SES and SSS were related to the tendency to perceive the world in a holistic way, or whether the focus on specific details as social cognitive models of social class postulate related differences in psychological tendencies. Interestingly, we found that people with a high objective SES tended to perceive the world in a more holistic way than people with low objective SES—*r* (3378) = 0.086, *p* (two-tailed) < 0.001—whereas SSS had a negative nonsignificant correlation with this psychological tendency: *r* (3342) = −0.027, *p* (two-tailed) = 0.119.

### Nonregistered Analyses

To further examine the validity of our conceptualization of objective SES, we computed Spearman’s rank correlation between educational attainment and gainful employment. The indicators merely had a small positive correlation—*r*_*s*_ (5289) = 0.150, *p* < 0.001—which suggests a low reliability of the score for objective SES. Additionally, we computed the correlation between objective SES and SSS and found a small correlation: *r* (3372) = 0.240, *p* < 0.001. This result indicates that people’s perceived standing in society was determined by more factors than their objective living conditions.

## Discussion and Concluding Remarks

Political bandwagon effects have received considerable attention in past research. Previous findings are, however, decidedly inconclusive (Barnfield 2020). The present study improves on prior research in several aspects. First, we integrated theories from political science and social psychology to introduce social class as a possible moderator of bandwagon effects. Second, we investigated this hypothesized moderation in a multiparty system. Third, we relied on high-quality, representative RCS data preceding an exceptionally strongly contested election in Germany. Fourth, we employed a rigorous methodological approach based on adequate modeling of the time-dependency of the data as well as preregistered analyses.

We found only limited evidence for bandwagon effects. Merely for the SPD, a higher vote share in polls (i.e., CMVI) was associated with a higher voting intention 1 day after polls were published. Supporting the notion of a bandwagon effect, this association was stronger for voters reporting to have seen polls previously. Contradicting our hypothesis, there was no evidence for moderation of the association by voters’ objective SES for any of the parties. In contrast to previous findings on consumer decisions, social class was not related to the tendency to follow a perceived majority regarding voting intentions. Exploratory analyses with different time lags or with SSS as indicator of social class resulted in the same pattern of results. However, for Alliance 90/the Greens we found first evidence that the association between CMVI and voting intention was greater for voters with less educational attainment.

### Limitations and Future Research

Although we did not find moderation of the association between CMVI and voting intentions by social class, our results provide important insights into bandwagon effects in multiparty systems as well as social class effects in the German context. Thus, our results can serve as guidance for fruitful future research in this field.

Looking at the results, it becomes clear that voting intentions as measured in the RCS data showed little variation across field days. From a statistical perspective, this makes it unlikely to find potential bandwagon effects. This finding is surprising because the 2021 election was characterized by a dynamic election campaign (e.g., Grahn and Süßmann 2021) and comparably volatile poll results. The dynamic of the poll results was, however, not reflected in the pattern of voting intentions from the RCS data (except for the SPD). There are methodological aspects that should be taken into account to understand this result.

Specifically, there were differences in the way the voting intention was assessed in the RCS data and the way the CMVI had been presented by the polling institutes. Importantly, polls did not report on the share of people who did not plan to cast a vote. We, however, included these participants in our assessment of voting intentions because bandwagon effects could potentially also affect them. Additionally, most polling institutes published so-called projected vote shares instead of raw data. Both aspects could have decreased the association between CMVI and measured voting intentions.

Furthermore, the absence of bandwagon effects might be linked with the investigated time period. Previous social psychological research has shown that majority influence is especially strong when people feel a high level of uncertainty (Deutsch and Gerard 1955). Because we investigated poll effects briefly before the election, most respondents had established their preferences already, which might have reduced the tendency to follow others’ opinions.

To summarize, our results indicate that even though the investigated election apparently provided optimal circumstances, bandwagon effects did not occur for the majority of parties in Germany. This implies that most voters did not conform to impersonal social influences exerted by poll results but arrived at their voting intention based on factors such as party identification, issue orientation, and candidate orientation (see Table A3). For these parties, public opinion did not impinge on itself.

Nevertheless, we found a positive relationship between the CMVI and measured voting intentions for the SPD. Looking at the pattern of the published poll results gives further insights (see online attachment, Figs. A1–A6): The SPD was the party with the largest increase in projected vote share during the 55 days before the election across the different polling institutes. Additionally, the party was leading the polls for approximately the last month before the election. During the investigated time span, poll results for the FDP, the Left, and AfD were relatively stable, whereas Alliance 90/the Greens and especially the CDU/CSU lost ground continuously. Accordingly, a bandwagon effect, which is defined as voters switching to a party perceived as the majority choice (Barnfield 2020; Schmitt-Beck 2015), is most plausible for the SPD.^19^ This result is in line with the findings by Faas et al. (2008), who identified a bandwagon effect for the SPD in the German federal election of 2005. Importantly, falling poll figures did not accelerate the downward trend for Alliance 90/the Greens or the CDU/CSU.

At the same time, the results provide insights into the factors determining the perception of a “winner” in poll results, which has been considered central to bandwagon effects. In the beginning of the investigated time span, the SPD probably did not appear to be a winner to most voters, as the CDU/CSU was leading the polls. This possibly changed when the poll results for the SPD started to rise and continued until the party took the lead in polls and remained at this position until the election. Thus, either the large increase in poll results over time or the leading position in polls (or a combination of both) might have elicited the perception of the SPD as the winner, which potentially motivated voters to switch to the winning side (Meffert et al. 2011; van der Meer et al. 2016). Because previous conceptualizations of a perceived winner were developed for two-party systems, they cannot be adequately applied to multiparty systems. Future research should further distinguish the characteristics of poll results eliciting bandwagon effects in multiparty systems.

However, we did not find evidence for the assumed causality direction of a bandwagon effect for the SPD, which limits the interpretability of the association between CMVI and voting intention. More concretely, voting intention was also associated with poll results published the day after the respective field day for the SPD (see online appendix, Table A4). This finding may indicate that there is no bandwagon effect and that both measures simply reflect, for example, general swings of public opinion. However, it can also be explained by the fact that CMVI and measured voting intention generally reflect the same underlying core construct. Additionally, polls from the day later are based on data that were collected a few days earlier—that is, temporally close to the respective RCS field day. Thus, the found association does not necessarily prove that there is no (causal) bandwagon effect.

Furthermore, in the context of cross-lagged panel designs, researchers have developed the concept of “causal dominance” to interpret similar patterns of associations (e.g., Schuurman et al. 2016). Accordingly, the strongest association between variables measured at different measurement occasions is thought to be the “causally dominant” one, as it exerts the most important causal effect and drives the mechanism. Following this argument, our goal was to identify which of the paths between poll results and voting intentions was the “causally dominant” (i.e., the larger) one. Our robustness check showed that the association between CMVI and voting intention at *t* + 1 was not significantly larger than the association with voting intention at *t* − 1. However, this test was quite underpowered, as it was based on only six cases (i.e., the parties), which prevented us from identifying the causally dominant path. Future studies should further tap into the causality direction of bandwagon effects by using longitudinal data with multiple measurement occasions. This would allow for the implementation of complete cross-lagged panel designs and the test of causal dominance for poll effects on voting intentions one or more days later.

Regarding our proposed moderation, we found that the CMVI was associated with voting intentions of voters across the social class spectrum equally. There are different potential explanations for the lack of moderation of bandwagon effects by social class. First, social class effects on majority influence might depend on the national context. So far, most evidence for effects of social class on majority influence comes from the United States (e.g., Na et al. 2016; Stephens et al. 2011, 2007). Compared with the United States, Germany is characterized by a lower income inequality (OECD 2022). Assuming that inequality is related to the salience of social class differences (Schneider 2019), social class might have weaker effects on individuals’ self-concepts and, consequently, the tendency to follow a perceived majority in Germany compared with the United States. To ensure the generalizability of social class effects, future research could benefit from further cross-national replications of social class effects on majority influence.

Second, the type of decision might play a role in the investigated effects. Whereas people of lower social class show a greater tendency to follow the majority in consumer choices (e.g., Na et al. 2016; Stephens et al. 2011, 2007), this might not be transferable to voting decisions. Possibly, lower-class voters do not follow a perceived majority in the voting context because they perceive the majority of voters (of a potentially higher social class) as less indicative of their own political interests.

Third, the operationalization of objective SES did not include income level, which has been defined as a relevant indicator of social class alongside educational attainment (e.g., Kraus et al. 2012). Because of a lack of a more suitable measure of income in the RCS study, we used gainful employment status as a proxy. There are, however, some indications that this variable did not adequately capture income level. For example, employment status was only weakly correlated with educational attainment, as a relatively large number of people worked full time and had a low educational level. Additionally, it had a weaker association with SSS (*r* (3372) = 0.119, *p* < 0.001) than educational attainment did (*r* (3372) = 0.279, *p* < 0.001). Interestingly, investigating educational attainment separately suggested that effects of CMVI were greater among less educated voters for Alliance 90/the Greens. This finding can be interpreted as first evidence that aspects of objective SES play a role in the size of bandwagon effects and is in line with results by Schmitt-Beck (1996) for the German parliamentary election of 1990. However, this result should be interpreted cautiously, as poll results showed a declining trend over time for this party. To provide a complete picture of the role of social class in bandwagon effects, future research should include a measure of income level.

Finally, some questions regarding the underlying psychological mechanisms of bandwagon effects remain open because of the use of cross-sectional, correlational survey data. For example, it is unclear how voters’ interpretation of poll results is influenced when they are exposed to several polls over the course of a few weeks. Based on social cognitive research, a so-called cumulative redundancy bias (Alves and Mata 2019) might occur, suggesting that voters’ impressions about the winner in polls are biased by how the poll results develop over time. Additionally, it remains open how accurately voters remember currently perceived poll results in multiparty contexts. One reason for a lack of evidence for bandwagon effects might be that voters’ memory for published polls is biased such that they remember the results for some parties more accurately than others. This would also distort the effects of poll results on voting intentions. Assessing recalled poll results during the election campaign could help shed light on the psychological underpinnings of bandwagon effects. Furthermore, due to the use of cross-sectional data, we were not able to model time-varying individual effects. Again, we think that future research in this field would benefit from using longitudinal data.

## Conclusion

The present study adds to research on the political bandwagon effect in multiparty systems and the moderating effects of social class. We found limited evidence that published polls were associated with voting intentions in the German parliamentary election of 2021. Only for the SPD were higher poll results associated with higher voting intentions among those who had previously seen the polls. Voters’ social class could not be identified as a boundary condition of bandwagon effects. Consequently, taking into account this sociodemographic characteristic of voters does not appear to resolve the mixed evidence in this field of research. Aside from calling into question the generalizability of previously found social cognitive effects of social class across different contexts, our research helps to identify avenues for future research on bandwagon effects in multiparty systems.

## Supplementary Information

Online appendix including tables and figures from robustness checks

Data set: Preelection poll results as retrieved from Wahlrecht.de (2021)

## Footnotes

1 The authors used a multilevel modeling approach, a different selection of published poll results, and a different time-lag between the publishing of polls and the survey data collection.

2 The dataset and its documentation are available via the GESIS data archive after one-time registration.

3 For each polling institute, data were manually assembled into a Microsoft Excel sheet for processing in R.

4 The item was “Please imagine a ladder with 11 rungs to show where people in Germany stand. At the top—on rung 11—are the people with the most money, the highest education and the best jobs. At the bottom—on rung 1—are those with the least money, the lowest education and the worst jobs or no job. Where would you place yourself on the ladder? Please tell me on which rung—from 1 to 11—you think you stand in relation to other people in Germany in your current phase of life.” Unlike the other variables used, SSS was assessed in the RCS postelection survey. Approximately 63% of the respondents from the preelection survey also participated in the postelection survey.

5 This measure had some limitations. It did not allow us to draw further inferences on the source of the poll or the time point within the last week when respondents had seen the poll(s). Furthermore, respondents might have misremembered the poll results or exactly when they saw the poll.

6 The published poll results, however, are not raw marginals. The polling institutes use specific procedures to transform the raw data into distributions that look like plausible election results. However, these procedures are not revealed to the public (Wüst 2003).

7 In this case, group-mean centering means that respondents’ personal scores were centered around the mean of the respondents of the respective field day.

8 If this was not the case, we would still compute the further planned analyses, as level 2 variation could be suppressed by level 1 predictors and therefore increased once level 1 predictors were introduced into the model (cf. Hoffmann and Klein 2013).

9 The Michigan model is a dominant model for the explanation and prediction of voting behavior. Indeed, Hoffmann and Klein’s (2013) analyses based on the 2009 RCS data show that party identification, candidate orientation, and issue orientation were significant predictors of voting intention.

10 We argue that such a test constitutes a conservative check of the expected poll effects, as the RCS voting intention on *t* − 1 was assessed with a smaller temporal distance to the data collection by the polling institutes compared with the RCS voting intention on *t* + 1. This smaller temporal distance might have increased their correlation.

11 However, because of power concerns and the previously mentioned limitations of the variable capturing poll perception, we evaluated our hypothesis based on the two-way interaction between objective social class and CMVI.

12 Additionally, these other studies were conducted in the United States, had smaller sample sizes, and used an experimental manipulation of the perceived majority opinion.

13 As the tool did not permit us to conduct 1000 replications in one simulation, we ran the same simulation ten times with 100 replications each and computed the mean value of the power for the cross-level interaction effect, as recommended by Astivia et al. (2019).

14 A model including CMVI as single predictor showed a similar result for this main effect: β = 0.096, *SE* = 0.039, *OR* = 1.100, 95% CI [1.019, 1.188], *p* = 0.007.

15 All presented *p*-values are one-tailed (as preregistered) unless stated otherwise.

16 For these parties, models without objective SES CMVI also had no significant main effect on voting intention, all *p* > 0.05.

17 Since our main analyses produced insignificant results regarding the hypothesized interaction effect, we included the detailed results in the online supplement. These have also been made available on osf.io (https://osf.io/g6r7v/).

18 As the interaction of interest did not reach significance in any of the analyses, we abstained from conducting an additional analysis with weights.

19 Additionally, it should be noted that poll effects are less likely to be detected with only little variation in poll results across field days.
