# Supplementary material for: Jumping on the Bandwagon: The Role of Voters’ Social Class in Poll Effects in the Context of the 2021 German Federal Election
Source: Polit Vierteljahresschr. 2022 Aug 10;64(1):51–78. doi: 10.1007/s11615-022-00417-3 (PMC9364306; doi:10.1007/s11615-022-00417-3)
Supplement: Supplementary file 1 — Online appendix including tables and figures from robustness checks [file 11615_2022_417_MOESM1_ESM.docx]

Online Appendix

# Table A1. Results from all Multilevel Logistic Regression Models Predicting Voting Intention with Educational Attainment as Indicator of Social Class (Time Lag 1)

|  | CDU/CSU | | |  | SPD | | |  | FDP | | |  | Bündnis90/ DIE GRÜNEN | | |  | DIE LINKE | | |  | AfD | | | |
| --- | --- | --- | --- | --- | --- | --- | --- | --- | --- | --- | --- | --- | --- | --- | --- | --- | --- | --- | --- | --- | --- | --- | --- | --- |
|  | β |  | *SE* |  | β |  | *SE* |  | β |  | *SE* |  | β |  | *SE* |  | β |  | *SE* |  | β |  | *SE* |  |
| Intercept | -1.782 | *** | 0.04 |  | -1.569 | *** | 0.04 |  | -2.751 | *** | 0.075 |  | -1.644 | *** | 0.041 |  | -3.049 | *** | 0.07 |  | -3.192 | *** | 0.072 |  |
| CMVI | 0.039 |  | 0.039 |  | 0.097 | ** | 0.04 |  | 0.037 |  | 0.072 |  | 0.052 |  | 0.039 |  | 0.093 |  | 0.066 |  | -0.063 |  | 0.071 |  |
| Education | -0.105 | ** | 0.04 |  | -0.245 | *** | 0.039 |  | 0.282 | *** | 0.077 |  | 0.575 | *** | 0.051 |  | 0.156 | * | 0.079 |  | -0.258 | *** | 0.063 |  |
| CMVI x Education | -0.034 |  | 0.039 |  | -0.017 |  | 0.039 |  | -0.011 |  | 0.07 |  | -0.098 | * | 0.049 |  | 0.015 |  | 0.074 |  | 0.021 |  | 0.063 |  |

*Note.* *N* (level 2) = 55, *N* (level 1) = 5291 for each model. Educational attainment was contrast coded (-1 for low education, 0 for medium education, and 1 for high education). *SE* = Standard Error, CMVI = Current Majority’s Voting Intention.

The *p*-values for CMVI and the interaction are one-sided due to the directed hypotheses.

* *p* < .05, ** *p* < .01, *** *p* < .001.

# Table A2. Results from all Multilevel Logistic Regression Models Predicting Voting Intention with Gainful Employment Status as Indicator of Social Class (Time Lag 1)

|  | CDU/CSU | | |  | SPD | | |  | FDP | | |  | Bündnis90/ DIE GRÜNEN | | |  | DIE LINKE | | |  | AfD | | | |
| --- | --- | --- | --- | --- | --- | --- | --- | --- | --- | --- | --- | --- | --- | --- | --- | --- | --- | --- | --- | --- | --- | --- | --- | --- |
|  | β |  | *SE* |  | β |  | *SE* |  | β |  | *SE* |  | β |  | *SE* |  | β |  | *SE* |  | β |  | *SE* |  |
| Intercept | -1.789 | *** | 0.04 |  | -1.564 | *** | 0.04 |  | -2.738 | *** | 0.074 |  | -1.545 | *** | 0.038 |  | -3.04 | *** | 0.066 |  | -3.2 | *** | 0.079 |  |
| CMVI | 0.038 |  | 0.039 |  | 0.103 | ** | 0.04 |  | 0.022 |  | 0.069 |  | 0.028 |  | 0.038 |  | 0.084 |  | 0.066 |  | -0.079 |  | 0.073 |  |
| Employment | -0.157 | *** | 0.043 |  | -0.267 | *** | 0.037 |  | 0.182 | ** | 0.066 |  | 0.112 | ** | 0.037 |  | -0.167 | * | 0.066 |  | 0.224 | ** | 0.082 |  |
| CMVI x Employment | -0.03 |  | 0.042 |  | 0.035 |  | 0.037 |  | 0.074 |  | 0.061 |  | -0.019 |  | 0.036 |  | -0.073 |  | 0.065 |  | 0.054 |  | 0.074 |  |

*Note.* *N* (level 2) = 55, *N* (level 1) = 5291 for each model. Employment status was contrast coded (-1 for little to no gainful employment, 0 for part-time/short-time gainful employment, and 1 for full-time gainful employment).

*SE* = Standard Error, CMVI = Current Majority’s Voting Intention.

The *p*-values for CMVI and the interaction are one-sided due to the directed hypotheses.

* *p* < .05, ** *p* < .01, *** *p* < .001.

# Table A3. Results from all Multilevel Logistic Regression Models Predicting Voting Intention for the Political Parties Represented in the German Bundestag including Covariates (Time Lag 1)

|  | CDU/CSU | | |  | SPD | | |  | FDP | | |  | Bündnis90/ DIE GRÜNEN | | |  | DIE LINKE | | |  | AfD | | | |
| --- | --- | --- | --- | --- | --- | --- | --- | --- | --- | --- | --- | --- | --- | --- | --- | --- | --- | --- | --- | --- | --- | --- | --- | --- |
|  | β |  | *SE* |  | β |  | *SE* |  | β |  | *SE* |  | β |  | *SE* |  | β |  | *SE* |  | β |  | *SE* |  |
| Intercept | -2.995 | *** | 0.087 |  | -2.419 | *** | 0.073 |  | -4.04 | *** | 0.157 |  | -3.093 | *** | 0.109 |  | -4.225 | *** | 0.177 |  | -5.304 | *** | 0.299 |  |
| Gender | -0.124 | * | 0.059 |  | -0.091 |  | 0.051 |  | -0.168 | * | 0.082 |  | -0.085 |  | 0.056 |  | -0.005 |  | 0.114 |  | -0.201 |  | 0.158 |  |
| Age | 0.108 |  | 0.072 |  | -0.005 |  | 0.059 |  | 0.073 |  | 0.087 |  | -0.235 | *** | 0.061 |  | 0.152 |  | 0.124 |  | 0.099 |  | 0.174 |  |
| Political interest | -0.265 | *** | 0.071 |  | -0.069 |  | 0.062 |  | -0.028 |  | 0.092 |  | -0.021 |  | 0.071 |  | 0.036 |  | 0.136 |  | -0.589 | *** | 0.165 |  |
| Interest in campaign | 0.140 | * | 0.068 |  | 0.014 |  | 0.063 |  | 0.024 |  | 0.088 |  | -0.084 |  | 0.071 |  | 0.084 |  | 0.132 |  | 0.479 | *** | 0.137 |  |
| Party identification | 1.100 | *** | 0.049 |  | 0.96 | *** | 0.04 |  | 0.553 | *** | 0.041 |  | 0.883 | *** | 0.043 |  | 0.735 | *** | 0.054 |  | 0.690 | *** | 0.074 |  |
| Issue orientation | 0.671 | *** | 0.045 |  | 0.546 | *** | 0.04 |  | 0.448 | *** | 0.044 |  | 0.829 | *** | 0.052 |  | 0.540 | *** | 0.059 |  | 0.554 | *** | 0.068 |  |
| Candidate orientation (1) | 0.745 | *** | 0.071 |  | 1.080 | *** | 0.079 |  | 1.317 | *** | 0.111 |  | 1.595 | *** | 0.102 |  | 0.858 | *** | 0.143 |  | 1.125 | *** | 0.127 |  |
| Candidate orientation (2) | 0.153 | * | 0.076 |  |  |  |  |  |  |  |  |  |  |  |  |  |  |  |  |  |  |  |  |  |

**Table A3 (continued)**

| CMVI | 0.016 |  | 0.056 |  | 0.141 | ** | 0.054 |  | 0.053 |  | 0.106 |  | 0.086 |  | 0.062 |  | 0.078 | ** | 0.111 |  | 0.072 |  | 0.163 |
| --- | --- | --- | --- | --- | --- | --- | --- | --- | --- | --- | --- | --- | --- | --- | --- | --- | --- | --- | --- | --- | --- | --- | --- |
| SES | -0.031 |  | 0.067 |  | -0.177 | ** | 0.058 |  | 0.202 | * | 0.095 |  | 0.170 | ** | 0.064 |  | 0.241 |  | 0.133 |  | 0.261 |  | 0.172 |
| CMVI x SES | -0.046 |  | 0.055 |  | -0.006 |  | 0.050 |  | 0.055 |  | 0.082 |  | -0.034 |  | 0.057 |  | -0.149 |  | 0.117 |  | 0.217 |  | 0.139 |
| *N* (level 2) | 4907 | | |  | 4933 | | |  | 4838 | | |  | 4936 | | |  | 3078 | | |  | 3593 | | |

*Note.* *N* (level 2) = 55. Candidate Orientation refers to the evaluation of the party’s top candidate and included ratings for two top candidates for DIE LINKE and AfD. For the CDU, candidate orientation (1) refers to Laschet and candidate orientation (2) refers to Merkel. *SE* = Standard Error, CMVI = Current Majority’s Voting Intention, SES = Objective Socio-Economic Status.

The *p-*values for CMVI and the interaction are one-sided due to the directed hypotheses.

* *p* < .05, ** *p* < .01, *** *p* < .001.

# Table A4. Results from all Multilevel Logistic Regression Models Predicting Voting Intention with CMVI Published One Day Later

|  | CDU/CSU | | |  | SPD | | |  | FDP | | |  | Bündnis90/ DIE GRÜNEN | | |  | DIE LINKE | | |  | AfD | | | |
| --- | --- | --- | --- | --- | --- | --- | --- | --- | --- | --- | --- | --- | --- | --- | --- | --- | --- | --- | --- | --- | --- | --- | --- | --- |
|  | β |  | *SE* |  | β |  | *SE* |  | β |  | *SE* |  | β |  | *SE* |  | β |  | *SE* |  | β |  | *SE* |  |
| Intercept | -1.791 | *** | 0.040 |  | -1.591 | *** | 0.041 |  | -2.763 | *** | 0.077 |  | -1.585 | *** | 0.040 |  | -3.022 | *** | 0.066 |  | -3.184 | *** | 0.071 |  |
| CMVI | 0.064 |  | 0.040 |  | 0.099 | ** | 0.041 |  | -0.011 |  | 0.074 |  | 0.045 |  | 0.040 |  | -0.052 |  | 0.066 |  | -0.060 |  | 0.070 |  |
| SES | -0.177 | *** | 0.041 |  | -0.345 | *** | 0.038 |  | 0.303 | ** | 0.068 |  | 0.356 | *** | 0.040 |  | -0.055 |  | 0.065 |  | -0.003 |  | 0.071 |  |
| CMVI x SES | -0.034 |  | 0.040 |  | -0.008 |  | 0.038 |  | -0.013 |  | 0.064 |  | -0.046 |  | 0.040 |  | -0.017 |  | 0.065 |  | -0.096 |  | 0.071 |  |

*Note.* *N* (level 2) = 53, *N* (level 1) = 5175 for each model. *SE* = Standard Error, CMVI = Current Majority’s Voting Intention, SES = Objective Socio-Economic Status.

The *p*-values for CMVI and the interaction are one-sided due to the directed hypotheses.

* *p* < .05, ** *p* < .01, *** *p* < .001.

# Table A5. Results from all Multilevel Logistic Regression Models Predicting Voting Intention including the Perception of Majority’s Voting Intention as Predictor (Time Lag 1)

|  | CDU/CSU | | |  | SPD | | |  | FDP | | |  | Bündnis90/ DIE GRÜNEN | | |  | DIE LINKE | | |  | AfD | | | |
| --- | --- | --- | --- | --- | --- | --- | --- | --- | --- | --- | --- | --- | --- | --- | --- | --- | --- | --- | --- | --- | --- | --- | --- | --- |
|  | β |  | *SE* |  | β |  | *SE* |  | β |  | *SE* |  | β |  | *SE* |  | β |  | *SE* |  | β |  | *SE* |  |
| Intercept | -1.794 | *** | 0.040 |  | -1.591 | *** | 0.041 |  | -2.775 | *** | 0.077 |  | -1.592 | *** | 0.040 |  | -3.041 | *** | 0.066 |  | -3.186 | *** | 0.076 |  |
| CMVI | 0.030 |  | 0.040 |  | 0.094 | * | 0.041 |  | 0.021 |  | 0.074 |  | 0.038 |  | 0.040 |  | 0.093 |  | 0.066 |  | -0.072 |  | 0.07 |  |
| SES | -0.172 | *** | 0.041 |  | -0.349 | *** | 0.037 |  | 0.249 | *** | 0.065 |  | 0.358 | *** | 0.040 |  | -0.039 |  | 0.066 |  | 0.011 |  | 0.076 |  |
| CMVI Perception | 0.102 | * | 0.041 |  | 0.186 | *** | 0.042 |  | 0.124 |  | 0.067 |  | 0.140 | ** | 0.043 |  | -0.023 |  | 0.066 |  | 0.050 |  | 0.075 |  |
| CMVI x SES | -0.029 |  | 0.041 |  | 0.006 |  | 0.038 |  | 0.060 |  | 0.062 |  | -0.056 |  | 0.039 |  | -0.044 |  | 0.065 |  | 0.056 |  | 0.072 |  |
| CMVI x CMVI Perception | 0.053 |  | 0.040 |  | 0.091 | * | 0.041 |  | -0.022 |  | 0.067 |  | -0.018 |  | 0.041 |  | 0.076 |  | 0.066 |  | 0.027 |  | 0.072 |  |
| SES x CMVI Perception | -0.036 |  | 0.040 |  | -0.011 |  | 0.039 |  | 0.192 | ** | 0.06 |  | -0.021 |  | 0.042 |  | -0.141 | * | 0.066 |  | -0.039 |  | 0.076 |  |
| CMVI x SES x CMVI Perception | -0.050 |  | 0.039 |  | 0.002 |  | 0.039 |  | 0.011 |  | 0.064 |  | -0.011 |  | 0.040 |  | -0.012 |  | 0.066 |  | -0.054 |  | 0.071 |  |

*Note.* *N* (level 2) = 55, *N* (level 1) = 5271 for each model. CMVI perception was dummy-coded (0 = no, 1 = yes). *SE* = Standard Error, CMVI = Current Majority’s Voting Intention, SES = Objective Socio-Economic Status.

The *p*-values for CMVI and the interaction are one-sided due to the directed hypotheses.

* *p* < .05, ** *p* < .01, *** *p* < .001.

# Table A6. Results from all Multilevel Logistic Regression Models Predicting Voting Intention for a Time Lag of Zero Days and Two Days

|  | CDU/CSU | | |  | SPD | | |  | FDP | | |  | Bündnis90/ DIE GRÜNEN | | |  | DIE LINKE | | |  | AfD | | | |
| --- | --- | --- | --- | --- | --- | --- | --- | --- | --- | --- | --- | --- | --- | --- | --- | --- | --- | --- | --- | --- | --- | --- | --- | --- |
|  | β |  | *SE* |  | β |  | *SE* |  | β |  | *SE* |  | β |  | *SE* |  | β |  | *SE* |  | β |  | *SE* |  |
| Lag 0 |  |  |  |  |  |  |  |  |  |  |  |  |  |  |  |  |  |  |  |  |  |  |  |  |
| Intercept | -1.790 | *** | 0.040 |  | -1.581 | *** | 0.040 |  | -2.749 | *** | 0.074 |  | -1.583 | *** | 0.039 |  | -3.023 | *** | 0.065 |  | -3.164 | *** | 0.070 |  |
| CMVI | 0.054 |  | 0.039 |  | 0.109 | ** | 0.041 |  | -0.061 |  | 0.069 |  | 0.057 |  | 0.039 |  | -0.022 |  | 0.065 |  | -0.123 |  | 0.069 |  |
| SES | -0.175 | *** | 0.041 |  | -0.343 | *** | 0.037 |  | 0.280 | *** | 0.066 |  | 0.356 | *** | 0.040 |  | -0.057 |  | 0.065 |  | -0.011 |  | 0.070 |  |
| CMVI x SES | 0.007 |  | 0.040 |  | 0.021 |  | 0.037 |  | 0.031 |  | 0.058 |  | -0.056 |  | 0.038 |  | 0.025 |  | 0.065 |  | 0.028 |  | 0.068 |  |
|  |  |  |  |  |  |  |  |  |  |  |  |  |  |  |  |  |  |  |  |  |  |  |  |  |
| Lag 2 |  |  |  |  |  |  |  |  |  |  |  |  |  |  |  |  |  |  |  |  |  |  |  |  |
| Intercept | -1.789 | *** | 0.040 |  | -1.581 | *** | 0.041 |  | -2.751 | *** | 0.073 |  | -1.582 | *** | 0.040 |  | -3.024 | *** | 0.065 |  | -3.160 | *** | 0.070 |  |
| CMVI | 0.016 |  | 0.039 |  | 0.105 | ** | 0.040 |  | -0.076 |  | 0.067 |  | 0.002 |  | 0.039 |  | -0.064 |  | 0.066 |  | -0.001 |  | 0.069 |  |
| SES | -0.175 | *** | 0.041 |  | -0.343 | *** | 0.037 |  | 0.279 | *** | 0.065 |  | 0.355 | *** | 0.039 |  | -0.057 |  | 0.065 |  | -0.012 |  | 0.070 |  |
| CMVI x SES | -0.014 |  | 0.041 |  | 0.014 |  | 0.037 |  | 0.092 |  | 0.059 |  | -0.052 |  | 0.039 |  | 0.002 |  | 0.065 |  | 0.084 |  | 0.068 |  |

*Note.* *N* (level 2) = 55, *N* (level 1) = 5291 for each model. *SE* = Standard Error, CMVI = Current Majority’s Voting Intention, SES = Objective Socio-Economic Status.

The *p*-values for CMVI and the interaction are one-sided due to the directed hypotheses.

* *p* < .05, ** *p* < .01, *** *p* < .001.

# Table A7. Results from all Multilevel Logistic Regression Models Predicting Voting Intention with Subjective Social Status as Indicator of Social Class (Time Lag 1)

|  | CDU/CSU | | |  | SPD | | |  | FDP | | |  | Bündnis90/ DIE GRÜNEN | | |  | DIE LINKE | | |  | AfD | | | |
| --- | --- | --- | --- | --- | --- | --- | --- | --- | --- | --- | --- | --- | --- | --- | --- | --- | --- | --- | --- | --- | --- | --- | --- | --- |
|  | β |  | *SE* |  | β |  | *SE* |  | β |  | *SE* |  | β |  | *SE* |  | β |  | *SE* |  | β |  | *SE* |  |
| Intercept | -1.768 | *** | 0.049 |  | -1.561 | *** | 0.046 |  | -2.803 | *** | 0.094 |  | -1.386 | *** | 0.047 |  | -3.088 | *** | 0.098 |  | -3.29 | *** | 0.099 |  |
| CMVI | 0.023 |  | 0.049 |  | 0.082 | * | 0.046 |  | -0.071 |  | 0.086 |  | 0.072 |  | 0.047 |  | 0.098 |  | 0.089 |  | -0.066 |  | 0.091 |  |
| SSS | 0.159 | ** | 0.049 |  | -0.078 |  | 0.045 |  | 0.227 | * | 0.092 |  | 0.138 | ** | 0.044 |  | -0.332 | *** | 0.088 |  | -0.066 |  | 0.094 |  |
| CMVI x SSS | -0.046 |  | 0.05 |  | -0.026 |  | 0.047 |  | 0.036 |  | 0.084 |  | -0.015 |  | 0.043 |  | -0.036 |  | 0.081 |  | 0.167 |  | 0.095 |  |

*Note.* *N* (level 2) = 55, *N* (level 1) = 3374 for each model. *SE* = Standard Error, CMVI = Current Majority’s Voting Intention, SSS = Subjective Social Status.

The *p*-values for CMVI and the interaction are one-sided due to the directed hypotheses.

* *p* < .05, ** *p* < .01, *** *p* < .001.

**Fig. A1**

Development of Voting Intention measured in the 2021 GLES RCS Survey and Poll Results for CDU/CSU from August 2 until September 25, 2021


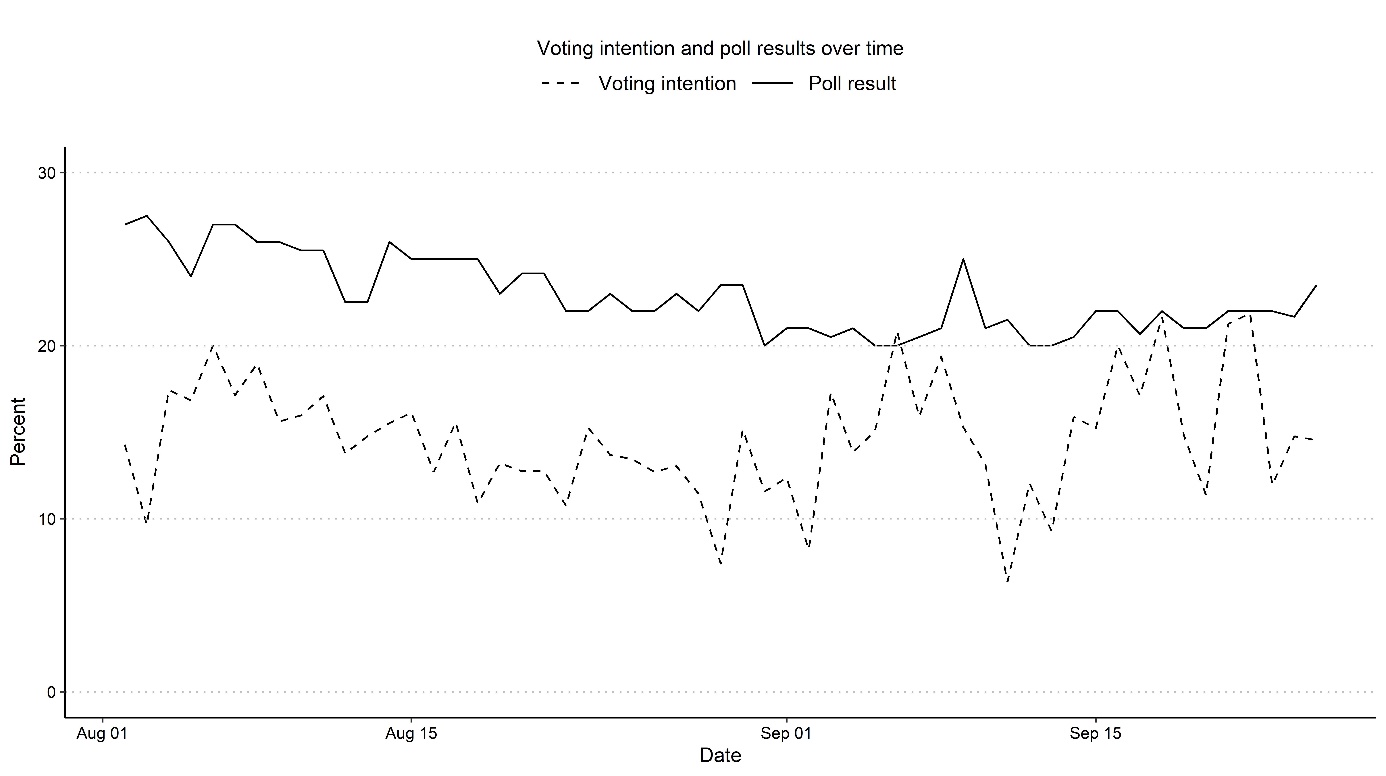


*Note.* *N* = 5291 respondents from the 2021 GLES RCS. Poll results are based on the polls by eight leading polling institutes in Germany.

**Fig. A2**

Development of Voting Intention measured in the 2021 GLES RCS Survey and Poll Results for SPD from August 2 until September 25, 2021


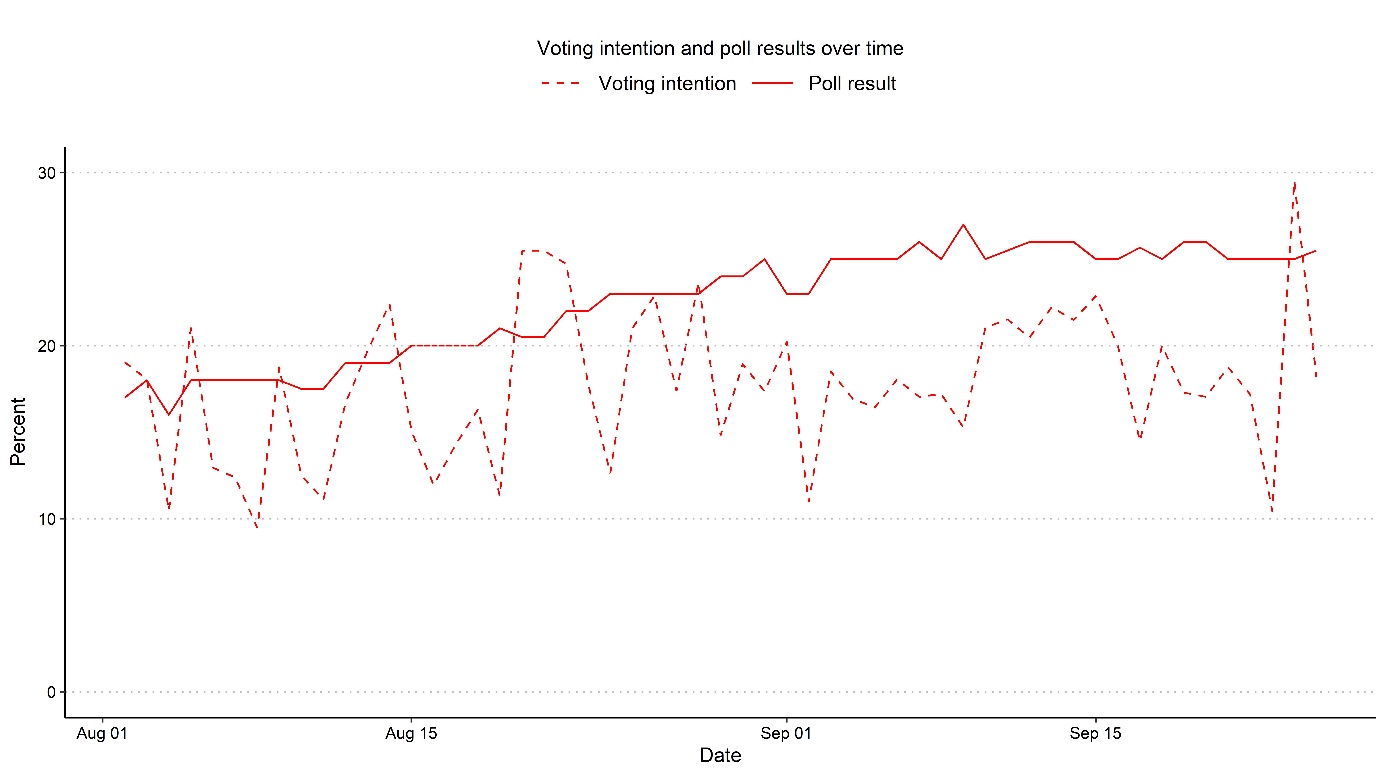

*Note.* *N* = 5291 respondents from the 2021 GLES RCS Survey. Poll results are based on the polls by eight leading polling institutes in Germany.

**Fig. A3**

Development of Voting Intention measured in the 2021 GLES RCS Survey and Poll Results for FDP from August 2 until September 25, 2021


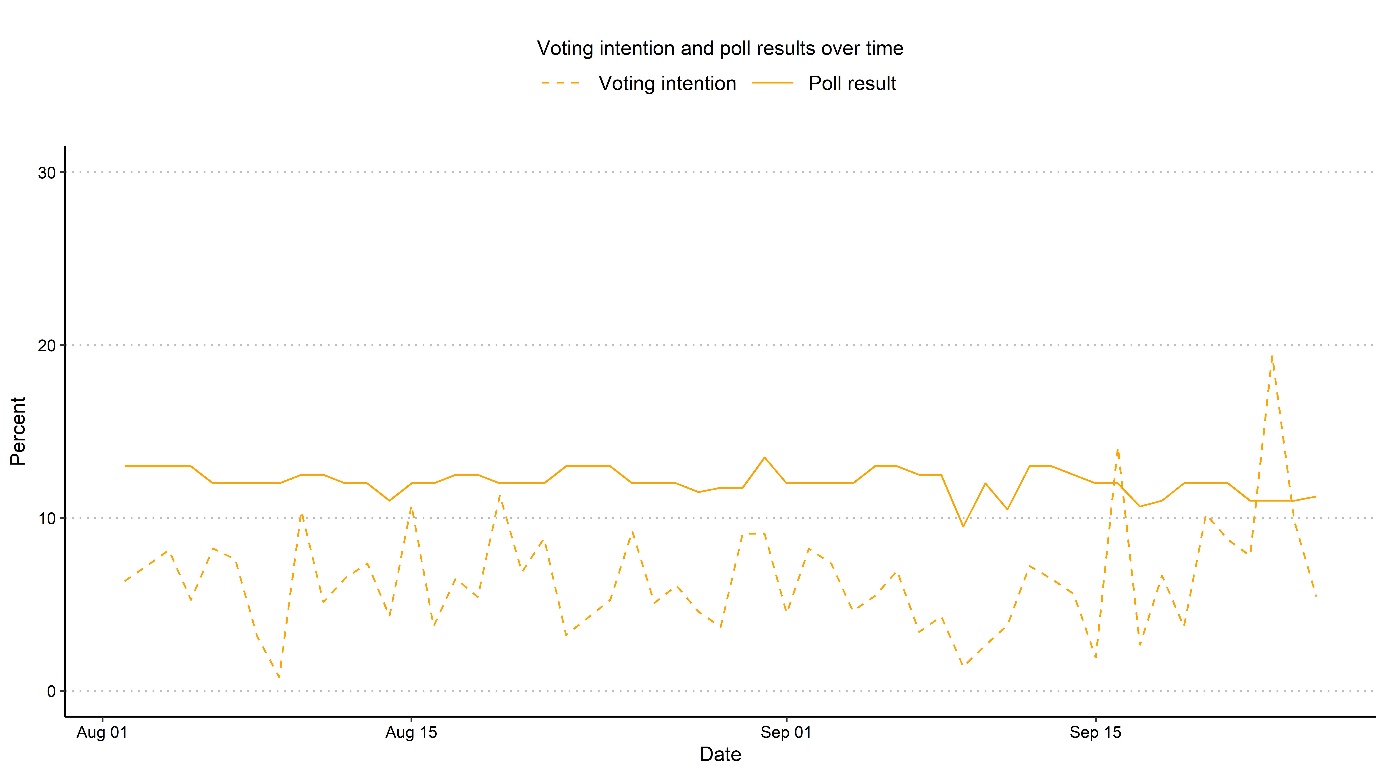


*Note.* *N* = 5291 respondents from the 2021 GLES RCS Survey. Poll results are based on the polls by eight leading polling institutes in Germany.

**Fig. A4**

Development of Voting Intention measured in the 2021 GLES RCS Survey and Poll Results for Bündnis90/DIE GRÜNEN from August 2 until September 25, 2021


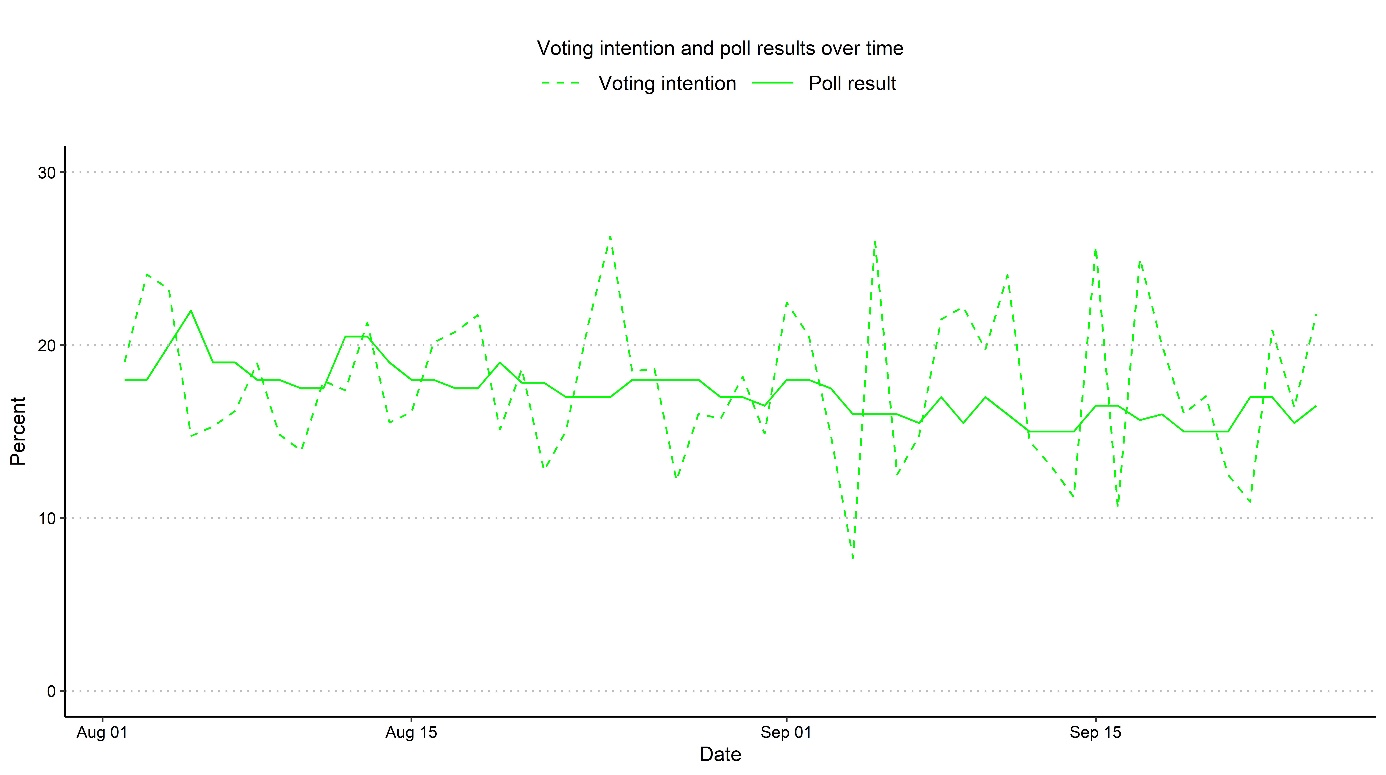
 *Note.* *N* = 5291 respondents from the 2021 GLES RCS Survey. Poll results are based on the polls by eight leading polling institutes in Germany.

**Fig. A5**

Development of Voting Intention measured in the 2021 GLES RCS Survey and Poll Results for DIE LINKE from August 2 until September 25, 2021


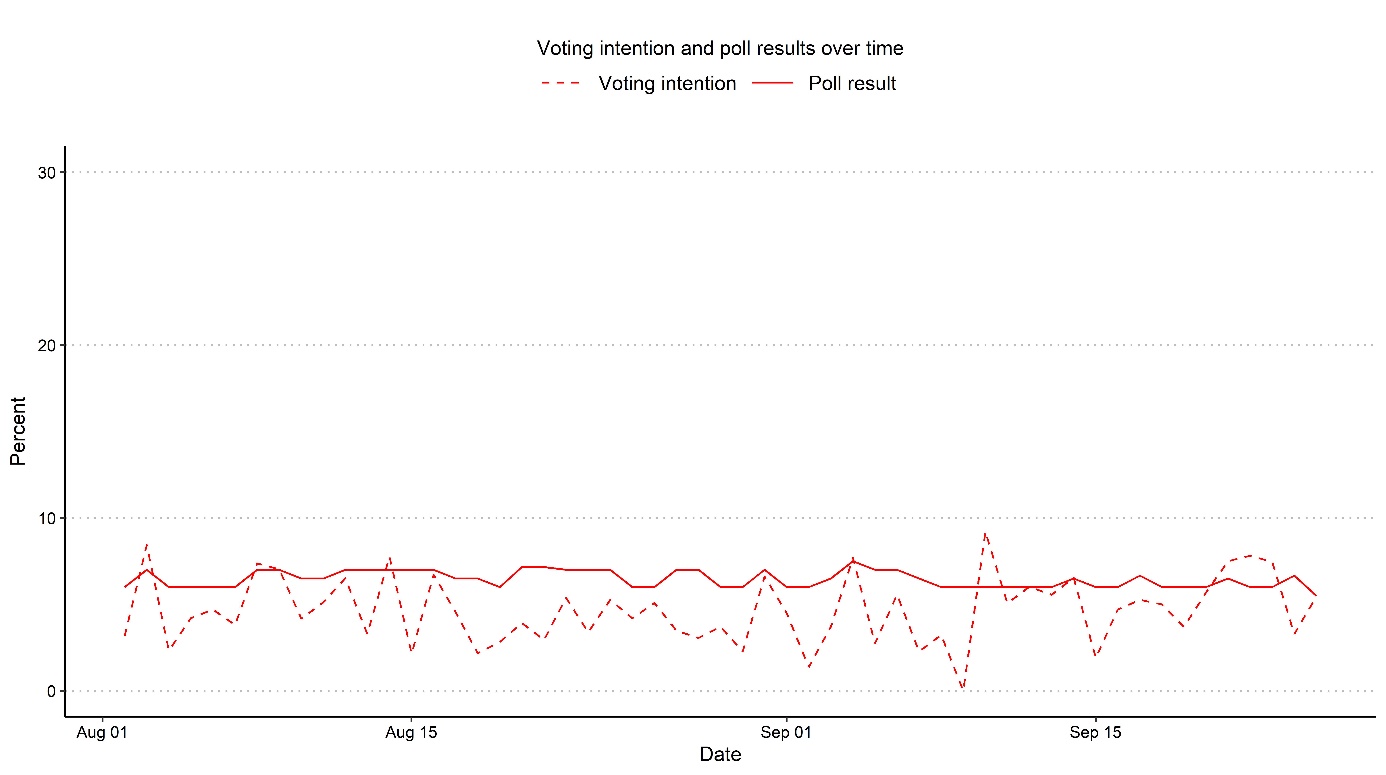


*Note.* *N* = 5291 respondents from the 2021 GLES RCS Survey. Poll results are based on the polls by eight leading polling institutes in Germany.

**Fig. A6**

Development of Voting Intention measured in the 2021 GLES RCS Survey and Poll Results for AfD from August 2 until September 25, 2021


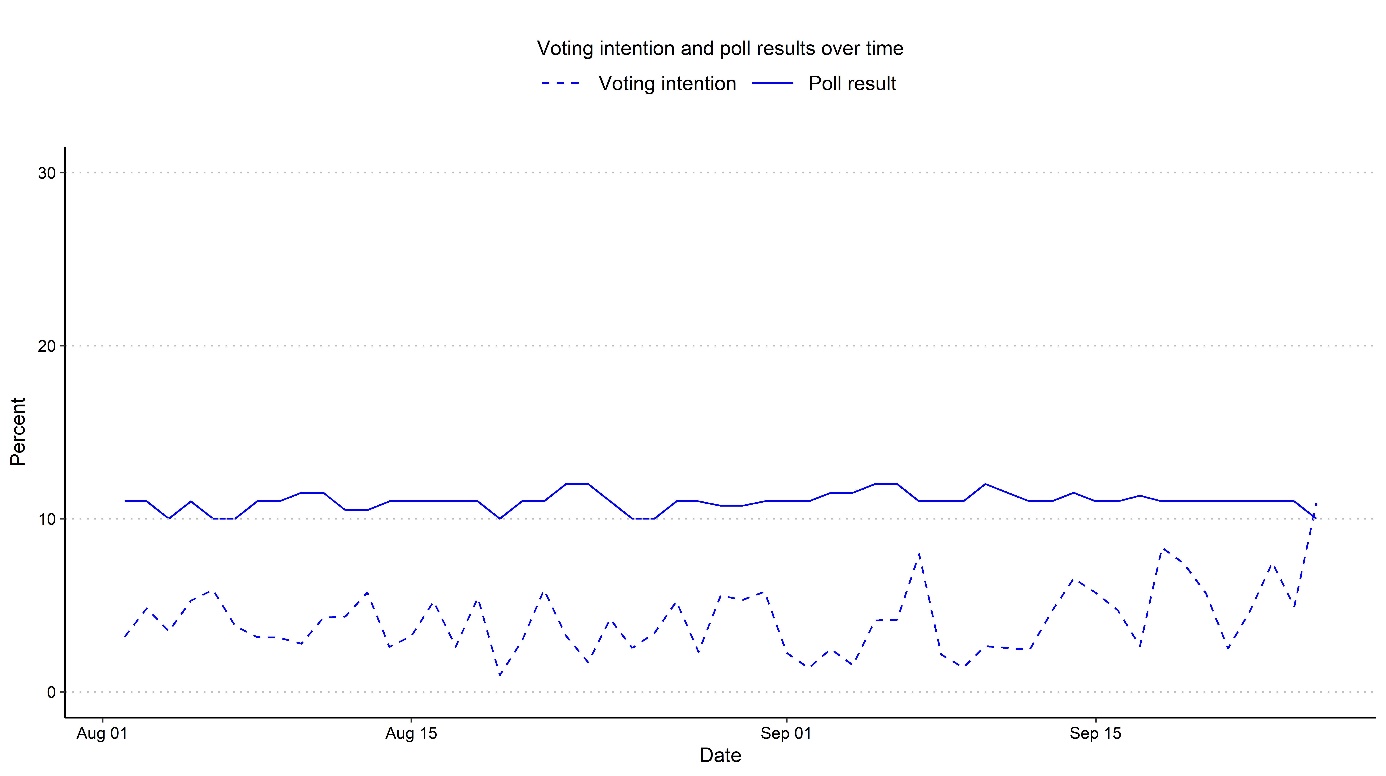
*Note.* *N* = 5291 respondents from the 2021 GLES RCS Survey. Poll results are based on the polls by eight leading polling institutes in Germany.
